# Hyaluronic Acid/Collagenase Ointment in the Treatment of Chronic Hard-to-Heal Wounds: An Observational and Retrospective Study

**DOI:** 10.3390/jcm11030537

**Published:** 2022-01-21

**Authors:** Francesco De Francesco, Marialuisa De Francesco, Michele Riccio

**Affiliations:** 1 Department of Reconstructive Surgery and Hand Surgery, AOU Ospedali Riuniti, Via Conca 71, 60126 Ancona, Italy; michele.riccio@ospedaliriuniti.marche.it; 2 Data Analysis Office, University of Milan, Via Colombo 46, 20133 Milan, Italy; Marialuisa.DeFrancesco@unimi.it

**Keywords:** chronic wounds, hyaluronic acid, collagenase, *Vibrio alginolitycus*, Bionect Start, Hyalo4 Start

## Abstract

Background: Wound bed preparation is an important concept in clinical practice and is related to adequate debridement. The use of proteolytic enzymes is an established method of enzymatic wound debridement, especially in hard-to-heal ulcers that are unresponsive to normal healing procedures and progress. The TIME framework (tissue, inflammation/infection, moisture balance, and edge of wound) offers an appropriate strategy to eliminate resistance to healing, as well as maximizing the healing process. Maintenance debridement, as opposed to sporadic debridement, may be proposed in preserving an adequate wound bed towards complete recovery. Collagenase has been effective in debridement due to its ability to degrade collagen and elastin. In this clinical context, collagenase taken from *Vibrio alginolitycus* is the most favorably expressed enzymatic debriding agent. Methods: This retrospective observational study evaluates the efficacy of an ointment based on hyaluronic acid and collagenase (Bionect Start^®^), considering a reduced healing time and greater healing quality. We included 70 patients with chronic wounds of different etiologies, including diabetes mellitus (20), post-traumatic ulcers (35), chronic burns of degrees I and II (10), and pressure ulcers (5). We analyzed wound characteristics using the wound bed score (WBS) concept, healing time, as well as operator and patient satisfaction. Results: Frequency of debridement efficacy in terms of wound bed cleansing varied from 26% after 2 weeks to 93% after 4 weeks. We observed complete healing in 62 patients within an eight-week period. The overall operator and patient satisfaction after 8 weeks were 100% and 90%, respectively. Moreover, all patients reported less pain. Conclusions: A combined action of hyaluronic acid and collagenase ointment demonstrated a reduction in healing time while improving healing quality, with a decrease in pain.

## 1. Introduction

**Figure 1:**
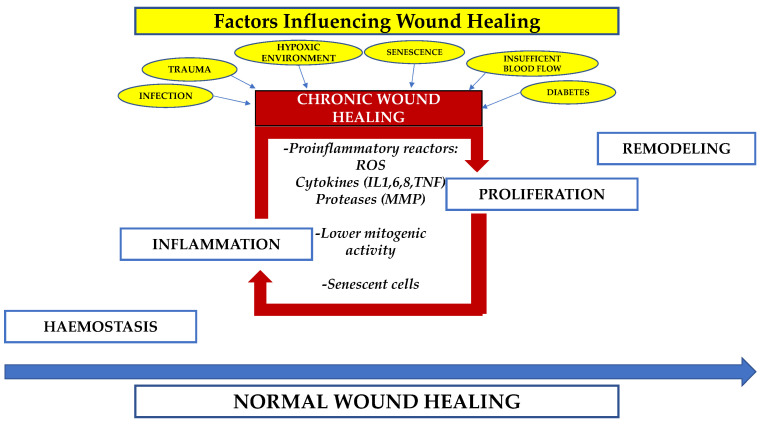
Phases of normal and chronic wound healing processes. Different conditions may influence normal wound healing turning into a chronic state, characterized by perpetuating an inflammatory and proliferative phase and promoting the release of inflammatory factors. (ROS: reactive oxygen species); (IL: interleukine); (TNF: tumor necrosis factor).

Wound healing is a complex biological repair procedure commonly developing in four concurring stages, including hemostasis, inflammation, proliferation and remodeling [1]. This elaborate and precarious procedure is moderately effective but may degenerate, causing chronic wound (CW) conditions, which are unresponsive to a natural biological healing mechanism. Such CW may remain at a proliferative or inflammatory state of wound healing [2] and typically revert to an inflammatory state [3] due to specific biochemical, microbial, and cellular abnormalities that interfere with healing progression (Figure 1).

The mechanism concurring to CW varies considerably but involves metabolic diseases (such as diabetes) [4], factors influencing blood supply (peripheral vascular disease) [5], elderly age [6], peripheral neuropathy [7], infection [8], malnutrition [9], altering of immune function, medications, or previous local tissue injury (trauma or radiation therapy). Moreover, external factors such as sustained pressure, temperature, and moisture also play an important role in enabling a wound to heal [10]. Although the etiology differs, CW are characterized by excessive levels of proinflammatory cytokines, proteases, reactive oxygen species, senescent cells, and a deficiency of functional stem cells [11,12].

A retrospective study conducted in 2018 reported that approximately 8.2 million people worldwide were affected by infected or non-infected wounds, which lead to hard-to-heal ulcers. Furthermore, significant interest and demand has emerged regarding wound care dressings, above all in the USA and Europe, currently representing the two largest markets in the world. Indeed, in 2014, the annual cost of wound care was approximately USD 2.8 billion; in 2021, spending increased to USD 3.5 billion [13]. The spending forecast is estimated at around USD 15 billion for 2022 and USD 22 billion for 2024 [14]. These costs highlight the importance of designing appropriate outpatient instruments to enhance healing. For this reason, Falanga and colleagues developed the concept of wound bed preparation [15,16]. Wound bed preparation is crucial for CW due to the difficult management of such atypical wounds [17]. This preparation aims to remove healing resistance as well as enhancing healing mechanisms with the formation of a stable wound, containing appropriate granulation tissue and a well-vascularized wound bed, providing the oxygen required to improve the ulcer [18]. The first and most important intervention is the debridement process, which is normally considered a simple removal of necrotic and infected tissue [19]. Additionally, debridement is applied to maintain an optimal uninfected wound bed, essential for the cutaneous regeneration process [20]. Two methods of debridement are herein described: surgical and enzymatic. Surgical debridement is commonly used for large surface wounds that are severely infected and often require the removal of exposed infected noble structures (bones, cartilage, and tendons). This debridement is more rapid and effective compared to other complementary methods, such as VAC and OTI therapy [21,22], even though it is more invasive. 

Enzymatic debridement is often conducted in smaller wounds not requiring surgical intervention or wounds that necessitate such a selective microenvironment [23], able to identify devitalized tissues only. It consists of the topical use of natural products, such as proteolytic enzymes or proteinases, whose activity is useful to eliminate necrotic tissue through a digestive mechanism of the not-vital tissue, but also to promote cell migration responsible for skin regeneration at wound bed level [24]. Several enzymatic debridement agents have been proposed such as trypsin [25], streptokinase–streptodornase combination [26], and subtilisin [27]. Bromelain is under investigation as an enzymatic debriding agent consisting of endopeptidases and other enzymes [28] that derive from the pineapple. However, the two most used proteolytic agents are papain and collagenase [29]. The application of both ointments (Accuzyme^®^ (Healthpoint, Fort Worth, TX, USA) and Noruxol^®^ (Smith&Nephew, Agrate Brianza (MB), Italy), respectively) has been incorporated into the treatment regimen of burn injuries and CW with beneficial effect. Papain, derived from Carica papaya, is part of nonselective preparations [30], while collagenase, derived from *Clostridium histolyticum,* is a selective preparation [31]. Collagenase has been successfully adopted for the degradation of collagen and elastin but was reported irrelevant in fibrin. Papain–urea is mainly used to solubilize fibrin. Investigations have revealed its safety and effectiveness in the debridement of cutaneous ulcers, and it is favorably applicable in long-term care and home care contexts. 

Although the most widely used collagenase preparation is derived from *Clostridium histolyticum* [31], in 2011, researchers characterized a new collagenase, derived from high-purity *Vibrio alginolitycus* [32], with ability to hydrolyze collagen molecules and to preserve periwound skin. It seems to be more selective and less aggressive compared to the other one [33]. Moreover, its association with hyaluronic acid (HA) is apparently able to enhance the proliferative effects of the collagen peptides [34,35]. 

The purpose of our retrospective study is to assess the clinical effects concerning Bionect Start^®^ (Fidia Farmaceutici, Abano Terme (PD), Italy) ointment when treating chronic hard-to-heal wounds.

## 2. Materials and Methods

A single-center observational and retrospective study was conducted to assess the efficacy and safety of HA/collagenase (Bionect Start^®^, Hyalo4 Start^®^, Fidia Farmaceutici, Abano Terme (PD), Italy) ointment in relation to the recovery rate of chronic hard-to-heal wounds in patients treated between January 2017 and December 2020. The study was performed in compliance with the Declaration of Helsinki and the Guidelines for Good Clinical Practice. The study protocol was approved by the local Ethics Committee (396/2021). Demographic data, medical history and wound measurements were collected (Table 1). 

### 2.1. Patient Selection

This study included 70 patients (45 male and 25 female) with an average age of 59.2 years (32–85 years) affected by multifactorial hard-to-heal ulcers, i.e., diabetes mellitus (*n* = 20), post-traumatic ulcers (*n* = 35), chronic burns degrees I and II (not healed in 12 weeks) (*n* = 10), and pressure ulcers (*n* = 5). Patients with hard-to-heal wounds and presence of necrotic tissue were included. All patients underwent the same therapeutic protocol, including wound disinfection with Amukine Med, saline solution cleansing, and application of HA/collagenase (hyaluronic acid sodium salt 0.2% + collagenase) ointment to the wounds in a 2 mm thick layer. A sterile tongue blade was employed to spread the ointment preparation over the whole wound area. A barrier ointment was applied to the surrounding periwound area. The wound was covered with a non-adherent gauze and then with a secondary dressing. We applied dressings to the ulcers twice a week at our clinical center, and performed follow-up visits (photographs, wound characteristics, exudates, edges, size and periwound skin evaluation, and infections by bacterial swab) at V0 (pre-treatment), V1 (two weeks), V2 (four weeks), V3 (two months), and V4 (one year). Treatment was continued until the wound was completely clean and covered by granulating tissue. No advanced wound dressing was applied so as to avoid interference with healing time. Wounds were diagnosed as fully healed when the normal reepithelization process was complete. 

### 2.2. Assessment Methods

During each visit, wound size, wound bed, and wound quality were evaluated. Changes in wound size are difficult to measure by length and width due to the irregular shape of the wounds; hence, we measured the surface area in cm^2^ using squared and transparent paper on which we traced the wound area with indelible marker pens (Oxford Health NHS Foundation Trust guidelines). On wound closure, a second assessment was performed after 2 weeks to confirm complete healing of the wound, according to AHRQ recommendations. the wound bed score [36] was adopted to evaluate black eschar, eczema/dermatitis, depth, scarring (fibrosis), colour of wound bed, oedema, resurfacing epithelium, exudate amount (Table 2). 

Each parameter was assigned a score of 0, 1, or 2. The scores were determined considering 0 as a poor healing outcome and 14 as the best healing outcome. The scores were divided into 4 quartiles [16]. The percentage of necrotic area was calculated by dividing the necrotic area, expressed in cm^2^, by the total ulcer area, expressed in cm^2^, and the result was reported as a percentage [16]. The scores were computed, considering 76–100% as poor and 0–25% as the best healing score. Patients with successful debridement were considered among those with a percentage of 0–25% (necrotic area out of the total area). A global assessment of each patient (PTGA) was conducted with the participant required to express aesthetic satisfaction using a five-point Likert scale: “much worse than expected”, “worse than expected”, “as expected”, “better than expected”, or “much better than expected”. A clinical observer global assessment (COGA) was performed, and the investigator was required to evaluate wound status using a five-scale set: 1 = deterioration, 2 = no change, 3 = slight improvement, 4 = moderate improvement, or 5 = significant improvement. Furthermore, pain during dressing change and any undesired systemic or local effects were also evaluated and recorded. The pain was scored using the Visual Analogic Scale (VAS 0-10). Side effects were documented from study initiation to study termination. All adverse events were reported as separate events, including signs, symptoms, or morbidities, and were monitored until complete recovery or conclusive physical examination and assessment.

### 2.3. Statistical Analysis

Measurements were conducted in relation to the groups. Statistically significant differences were measured by the Student *t*-test. The threshold for statistical difference was based on the *p*-value, significant at <0.05. The statistical analysis software (SPSS) was used to conduct the two-tailed Student *t*-test along with the Kolmogorov–Smirnov test.

## 3. Results

Between January 2017 and December 2020, 70 patients were enrolled in the study. Of the total, 45 (64.3%) were male and 25 (35.7%) were female, with a mean age of 59.2 (range 32–85 years). Demographic data, medical history, and wound measurements were collected (Table 3). Wound healing was completed at V3 (over a two-month period) in 62 patients (87%) out of 70 subjects, and 100% complete wound healing was observed, with 0% recurrence after one year. A significantly higher wound recovery rate was observed at each evaluation compared to baseline: at V1 (100% vs. 74% (26% reduction); 95% confidence interval (CI) = 20.7–31.1%; *p* = 0.000), at V2 (100% vs. 6%; (94% reduction) 95% CI = 90–99.8; *p* = 0.000), at V3 (100% vs. 5.4%; (95% reduction) 95% CI = 91–98%; *p* = 0.000), and at V4 (100% vs. 0%; (100% reduction) 95% CI = 100–100%; *p* = 0.000).

**Figure 2:**
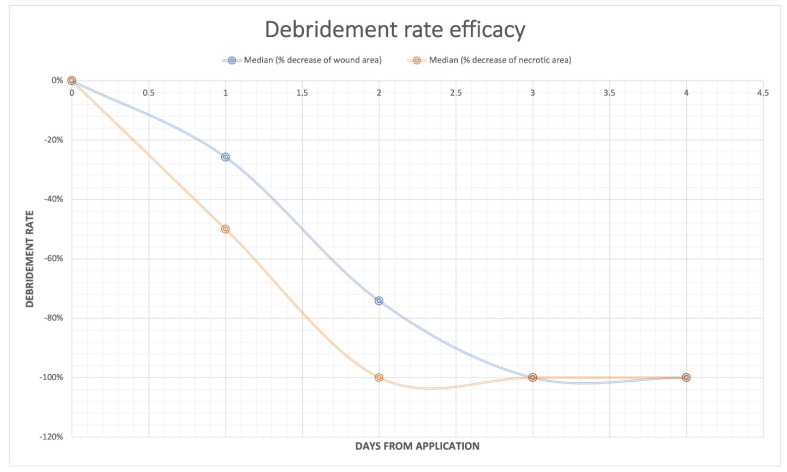
The rate of debridement efficacy. Trends of the percentage of necrotic area (orange line) and total ulcer area (blue line) during the treatment. Boxes represent the 95% confidence intervals.

The rate of debridement efficacy considering cleansing of the wound bed significantly increased during the treatment (*p* < 0.001). The total ulcer area significantly decreased at V2 (after week 4 of application) compared to V0 (baseline pre-treatment) (*p* = 0.000), while the necrotic area significantly decreased at V1 (after week 2) (*p* < 0.000). The necrotic component of the ulcer decreased at a higher rate compared to the total area (Figure 2).

A significant improvement was observed regarding qualitative parameters of the ulcer, such as black eschar, eczema/dermatitis, depth of scarring (fibrosis), color of wound bed, oedema, resurfacing epithelium, and exudate amount (Table 4).

**Figure 3:**
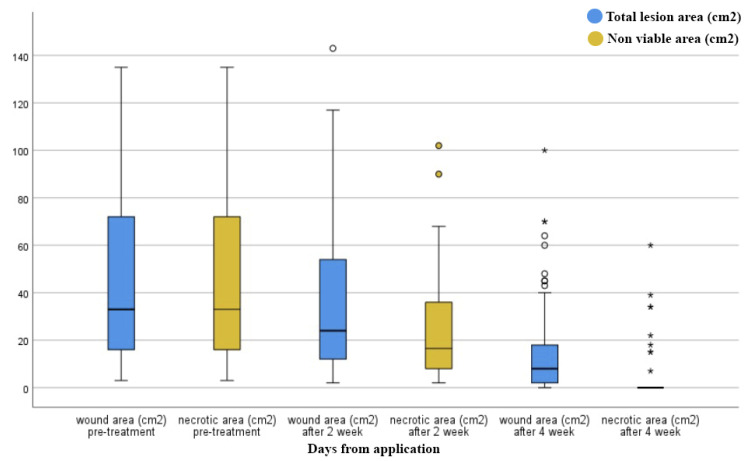
Box plots representing the evolution of total and necrotic area during the treatment. Boxes represent the 95% confidence intervals, the black line the median value, and whiskers are the lower and upper fence valuce. Circles and asterix identify observations that are smaller than the lower fence or larger than the upper fence.

A significant shift in wound healing progression was observed during treatment compared to baseline (Figure 3). Pain reduction after two months was remarkable in all patients, with a statistically significant reduction (*p* < 0.001) in pain experience. Moreover, a bacterial wound culture was performed with resulting positive V0 swabs in 60% of patients. Exudate was observed in 70% of patients. During the treatment, a progressive reduction in contaminated swabs and exudate was found compared to baseline (*p* < 0.001). At V0, analysis of wound exudate gave a positive result for Staphylococcus aureus (6 patients), Pseudomonas aeruginosa (10 patients), Enterococcus faecalis (4 patients), Bacillus cereus (2 patients), and for Klebsiella pneumoniae (1 patient). Infected wounds were treated after antibiogram results with specific oral antibiotics administration. At the end of the treatment, the swab culture analysis revealed the absence of bacterial pathogens. However, two lesions were positive for Enterococcus faecalis, and three lesions were positive for Pseudomonas aeruginosa.

**Figure 4:**
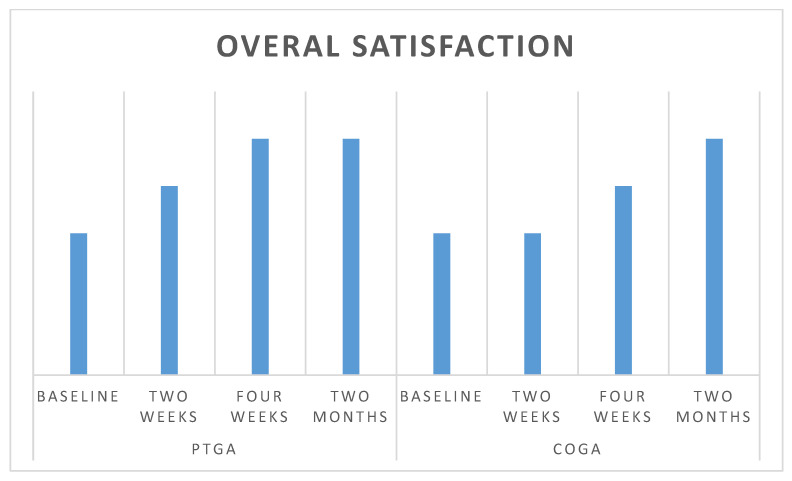
Average global assessment scale PTGA and COGA. The Likert 5 scale point did not show significant differences between investigator and patients in the different endpoint (V1–V4).

Further investigations were conducted to evaluate secondary objectives, specifically patient satisfaction regarding wound outcomes (PTGA) and clinician’s global assessment of the wound (COGA) over time (Figure 4).

**Figure 5:**
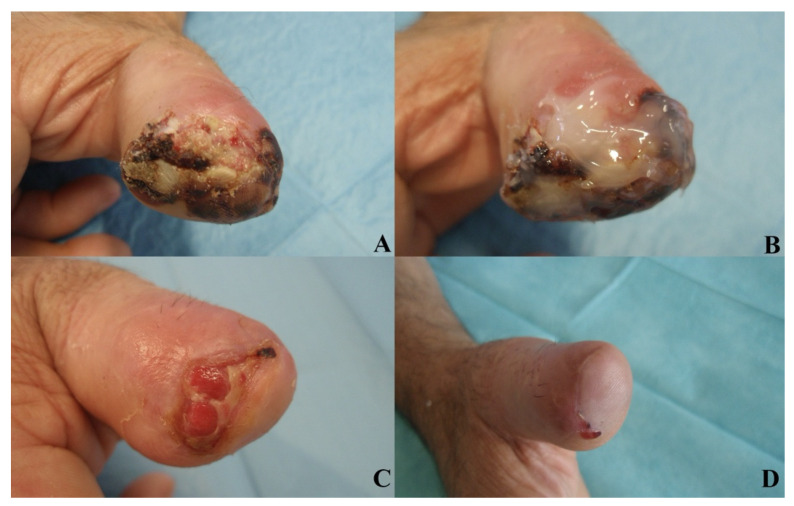
Post-traumatic case. AM, 45 y/o. Affected by hypertension, reported a chronic post-traumatic loss of substance (amputation) of the distal phalanx in the first finger of right hand. The aspect of the wound after injury (**A**). The application of Bionect Start ointment (**B**). Wound aspect at V1 (**C**) and final aspect with complete reepithelialization in V2 (**D**).

**Figure 6:**
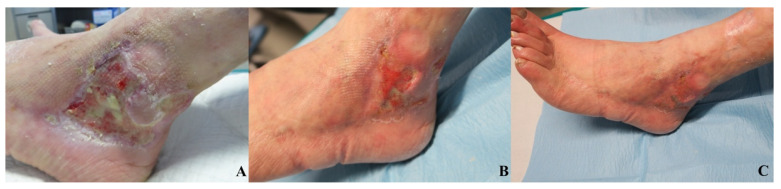
Vascular ulcer case. MR, 78 y/o. Affected by venous insufficiency, reported a chronic vascular loss of substance of lateral side of left ankle. The aspect of the wound pre-treatment (**A**). Wound aspect at V1 (**B**) and final aspect with complete reepithelialization in V2 (**C**).

**Figure 7:**
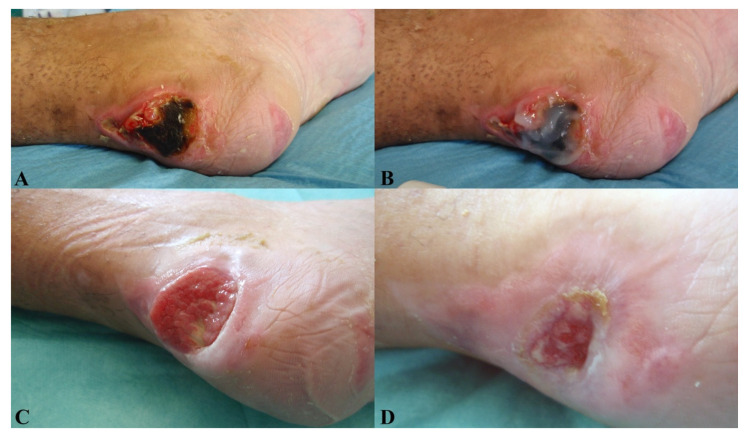
Diabetic case. PP, 65 y/o. Affected by diabetes, reported a chronic diabetic loss of substance of heel with necrosis of the superficial Achilles tendon. The aspect of the wound pre-treatment (**A**). The application of Bionect Start ointment (**B**). Wound aspect at V1 (**C**) and partial reepithelialization in V2, with a marked reduction in the ulcer area and necrosis (**D**).

No significant differences were observed in patient satisfaction and investigator evaluation. No side effects were reported in relation to the procedures or to the experimental product. Figure 5, Figure 6 and Figure 7 show examples of clinical cases.

## 4. Discussion

CW are indeed an arduous clinical and social issue, with 6.5 million people affected in the United States and 2 million people in Europe [37]. In the world’s largest wound-dressing regions, the United States and Europe, a significant demand for wound care products is evident. Globally, the annual cost for wound care was an average of USD 3.5 billion in 2021, which is expected to rise to USD 22 billion in 2024. Wound bed preparation plays a fundamental role in CW management with numerous debridement procedures being applied in clinical contexts [38]. Mechanical debridement is often a restrictive procedure due to the patient experiencing significant pain in order to achieve adequate wound cleansing, which must be performed in an operating room under anesthesia. For this reason, proteolytic enzymes have been successfully used in difficult ulcers [39], and enzymatic debridement is one current, non-invasive, painless method to remove necrotic tissue in long-term care. The most widely used enzyme in Europe is collagenase [40]. Collagenase is an ideal enzymatic debriding agent. Collagenase ointment has been favorably proposed as a selective enzymatic debriding agent, with current commercial use derived from the bacterial strain *Clostridium histolyticum* and from the bacterial strain *Vibrio alginolyticus* [33,41]. Reports from in vitro investigations have demonstrated that the *Vibrio alginolyticus* enzyme is as effective as *Clostridium histolyticum* on many types of collagens with equally active digestion of collagen type I. Conversely, *Vibrio alginolyticus* collagenase degrades fibronectin and decorin to a lesser extent than *Clostridium histolyticum* collagenase. This is an important insight, as decorin and fibronectin possess structurally important components of dermal extracellular matrix (ECM), and fibronectin is essential to dermal fibroblast migration within the wound-healing process [42]. The difference may be attributable to a higher selectivity of *Vibrio alginolyticus* collagenase, in which a completely active enzyme is observed on the collagen filaments binding the necrotic tissue to the wound bed. Conversely, the same collagenase remains inactive against crucial elements of the dermal ECM [33]. This response may explain the moderate effect of collagenase ointment from *Vibrio alginolyticus* on perilesional, healthy skin [16,41,43,44]. Several in vivo studies have demonstrated the effectiveness of *Vibrio alginolyticus* collagenase with a lower rate of perilesional skin complications compared with collagenase-based products derived from *Clostridum histolyticum*. Moreover, collagenase from *Vibrio alginolyticus* achieved an average inferior healing time compared to *Clostridium histolyticum*. Many authors have speculated that the combination of the fibrinolytic enzyme with a product able to stimulate tissue regeneration, such as HA, is a decisive factor in promoting rapid and effective healing of CW. Accordingly, in association with the specific action of the *Vibrio alginolyticus* enzyme, HA facilitates the recall of cells in the injured area and guides the deposition of ECM fibrous components, protecting the newly formed granulation tissue from oxygen-free radicals that impair wound healing [45].

This retrospective observational study has highlighted the importance and effectiveness of advanced bioactive dressings, such as Bionect Start^®^ ointment, in CW treatment. The application of Bionect Start^®^ promotes vascularized tissue and reduces formation of fibrin and exudate. This reduction is most likely due to a more specific presence of collagenase, accountable for fibrin and necrotic tissue lysis, and for macrophage engagement at the wound site and the consequent digestion of eschar and devitalized material at the wound bed.

We observed complete wound bed healing in 87% of patients in 2 months. On the other hand, some authors reported complete healing at the same endpoint in 27% of patients [33].

Total ulcer area significantly decreased after 4 weeks compared to baseline. This finding is in agreement with those found in the literature, compared to placebo [37].

Moreover, necrotic area significantly decreased after 2 weeks, as reported in the literature [45,46] and compared to mechanical debridement and *Clostridium hystoliticum* collagenase [33], with a statistically significant wound reduction over time.

The improved healing conditions may be in relation to hyaluronan in the ointment composition. This molecule maintains an optimal moist environment and promotes the healing process, as can be seen from our results, which show a clear reduction in discomfort, erythema, and swelling; the presence of hyaluronan might inhibit scar formation and yield more satisfactory outcomes [47]. Hyaluronan acid also exerts a protective action on wound edges through ECM matrix formation, modulation of the inflammatory reaction, and collagen deposition.

Moreover, pain reduction was significantly higher after 2 months compared to baseline, and no differences were found between patient satisfaction and clinics’ evaluation. These findings confirm the safety and efficacy of this ointment.

Despite the great results obtained, this retrospective, observational, and longitudinal study had some limitations. Firstly is the lack of a comparison arm, as previously performed [41,48]. A further limitation was the lack of in vitro analysis, such as histological and immune-histochemical evaluation, which enables the analysis of an authentic regenerative potential regarding Bionect Start^®^. In this regard, the authors are currently performing studies to actualize these points. Nevertheless, this study had a very long-term follow-up period, with a large sample of participants, allowing the observation of the absence of long-term recurrences.

## 5. Conclusions

In conclusion, based on this retrospective observational study, Bionect Start^®^ ointment shows a higher wound recovery rate over time and a significative reduction in total ulcer area and necrotic area compared to baseline. It displayed chemical, physical, and clinical potential to confirm easy operability in CW treatment and its role in protecting periwound skin. This is probably mainly due to collagenase association with HA, which allows better control of inflammation and modulates collagen deposition.

At the end of the study, all patients reached complete wound debridement. Furthermore, all other parameters associated with the ulcer, such as pain and patient satisfaction, showed significant improvement over time. No adverse events or hypersensitivity reactions were reported.

Further studies are necessary to prove the regenerative effect of Bionect Start^®^ ointment through an in vitro specific analysis and compared to in vivo outcomes. Moreover, perspective and randomized in vivo clinical trials are preferred.

## Figures and Tables

**Table 1:** Inclusion and exclusion criteria.

	Bionect Start
**Patients**	70
**Sex**	
Male	45 (65%)
Female	25 (35%)
**Age**	
Average	59.2
SD	12.76
Range	32–85
**Smokers**	
Yes	40 (57%)
No	30 (43%)
**BMI**	
Average	32.56
Median	31.7
SD	8.276
Range	19.9–81.6
**Etiologies**	
Diabetes	20 (28%)
Post-traumatic ulcers	35 (50%)
Burns	10 (14%)
Pressure ulcers	5 (7%)

**Table 2:** Wound bed score by Falanga, 2006.

	Scores of 0	Scores of 1	Scores of 2
**Black Eschar**	>25% of wound surface area	0–75%	>75% of wound surface area
**Eczema/Dermatitis**	Severe	Moderate	None/mild
**Depth/granulation**	Severely depressed or raised when compared to periwound skin	Moderate	Flushed or almost even
**Scarring**	Severe	Moderate	None/mild
**Color of wound bed**	None	50–75%	>75%
**Oedema/Swelling**	Severe	Moderate	None/mild
**Resurfacing epithelium**	None	25–75%	>75%
**Exudate amount**	Severe	Moderate	None/mild

**Table 3:** Descriptive statistics of ulcer areas over time and results obtained after classifying patients according to the 5-point scale of debridement.

		Time of Visit				
		Pre-Treatment (V0)	2 Weeks (V1)	4 Weeks (V2)	2 Months (V3)	1 Year (V4)
Scale of debridement	1	0	0	61 (87%)	62 (89%)	70 (100%)
	2	0	0	0	5 (0.7%)	
	3	0	9 (13%)	7 (10%)	3 (0.4%)	
	4	0	33 (47%)	1 (0.1%)		
	5	70 (100%)	28 (40%)	1 (0.1%)		
Total area (cm^2^)		60 (3–576)	43 (2–360)	15 (0–100)	4 (0–50)	0
Necrotic area (cm^2^)		60 (3–576)	29 (2–210)	3 (0–60)	2 (0–30)	0
Percentage of necrotic area (%)		100	74 (33–100)	7 (0–87)	38 (0–71)	0

**Table 4:** Descriptive statistics of qualitative parameters of the ulcers with WBS score.

		Time of Visit			
	WBS Score	Pre-Treatment (V0)	2 Weeks (V1)	4 Weeks (V2)	2 Months (V3)
black eschar	0	46 (66%)	0 (0%)	0 (0%)	0 (0%)
	1	24 (34%)	39 (56%)	39 (56%)	0 (0%)
	2	0 (0%)	31 (44%)	31 (44%)	70 (100%)
eczema/dermatitis	0	18 (26%)	0 (0%)	0 (0%)	0 (0%)
	1	52 (74%)	26 (37%)	0 (0%)	0 (0%)
	2	0 (0%)	44 (63%)	70 (100%)	70 (100%)
depth scarring	0	39 (56%)	0 (0%)	0 (0%)	0 (0%)
	1	31 (44%)	37 (53%)	0 (0%)	0 (0%)
	2	0 (0%)	33 (47%)	70 (100%)	70 (100%)
color of WB	0	60 (86%)	31 (44%)	0 (0%)	0 (0%)
	1	10 (14%)	38 (54%)	24 (34%)	0 (0%)
	2	0 (0%)	1 (1%)	46 (66%)	70 (100%)
oedema	0	17 (24%)	3 (4%)	0 (0%)	0 (0%)
	1	30 (43%)	16 (23%)	3 (4%)	0 (0%)
	2	23 (33%)	51 (73%)	67 (96%)	70 (100%)
resurfacing epithelium	0	27 (39%)	0 (0%)	0 (0%)	0 (0%)
	1	43 (61%)	26 (37%)	0 (0%)	0 (0%)
	2	0 (0%)	44 (63%)	70 (100%)	70 (100%)
exudate amount	0	70 (100%)	0 (0%)	0 (0%)	0 (0%)
	1	0 (0%)	70 (100%)	0 (0%)	0 (0%)
	2	0 (0%)	0 (0%)	70 (100%)	70 (100%)

## Data Availability

The clinical data used to support the findings of this study are included within the article.

## Abbreviations

COGA	clinician’s global assessment of the wound
CW	chronic wound
ECM	extracellular matrix
HA	hyaluronic acid
PTGA	patient satisfaction regarding wound outcomes
VAS	visual analogic scale

## References

[1] Gilmore M.A. (1991). Phases of wound healing. Dimens. Oncol. Nurs..

[2] Han G., Ceilley R. (2017). Chronic Wound Healing: A review of current management and treatments. Adv. Ther..

[3] Demidova-Rice T.N., Hamblin M.R., Herman I.M. (2012). Acute and impaired wound healing: Pathophysiology and current methods for drug delivery, part 1: Normal and chronic wounds: Biology, causes, and approaches to care. Adv. Skin Wound Care.

[4] Pawar K.B., Desai S., Bhonde R.R., Bhole R.P., Deshmukh A.A. (2021). Wound with diabetes: Present Scenario and Future. Curr Diabetes Rev..

[5] Schneider C., Stratman S., Kirsner R.S. (2021). Lower extremity ulcers. Med. Clin. N. Am..

[6] Alam W., Hasson J., Reed M. (2021). Clinical approach to chronic wound management in older adults. J. Am. Geriatr. Soc..

[7] East J.M., Fray D.A., Hall D.E., Longmore C.A. (2015). Chronic neuropathic ulcer is not the most common antecedent of lower limb infection or amputation among diabetics admitted to a regional hospital in Jamaica: Results from a prospective cohort study. BMC Surg..

[8] Falcone M., De Angelis B., Pea F., Scalise A., Stefani S., Tasinato R., Zanetti O., Dalla Paola L. (2021). Challenges in the management of chronic wound infections. J. Glob. Antimicrob. Resist..

[9] Martinez Garcia R.M., Fuentes Chacon R.M., Lorenzo Mora A.M., Ortega Anta R.M. (2021). Nutrition in the prevention and healing of chronic wounds. Importance in improving the diabetic foot. Nutr. Hosp..

[10] Wilkinson H.N., Hardman M.J. (2020). Wound healing: Cellular mechanisms and pathological outcomes. Open Biol..

[11] Goldberg S.R., Diegelmann R.F. (2020). What Makes Wounds Chronic. Surg. Clin. N. Am..

[12] Frykberg R.G., Banks J. (2015). Challenges in the Treatment of Chronic Wounds. Adv. Wound Care.

[13] Nussbaum S.R., Carter M.J., Fife C.E., DaVanzo J., Haught R., Nusgart M., Cartwright D. (2018). An Economic Evaluation of the impact, cost, and medicare policy implications of chronic nonhealing wounds. Value Health.

[14] Sen C.K., Roy S. (2019). Socioeconomic approach to wound care: A new patient-centered paradigm. Adv. Wound Care.

[15] Panuncialman J., Falanga V. (2009). The science of wound bed preparation. Surg. Clin. N. Am..

[16] Falanga V., Saap L.J., Ozonoff A. (2006). Wound bed score and its correlation with healing of chronic wounds. Dermatol. Ther..

[17] Falanga V., Brem H., Ennis W.J., Wolcott R., Gould L.J., Ayello E.A. (2008). Maintenance debridement in the treatment of difficult-to-heal chronic wounds. Recommendations of an expert panel. Ostomy Wound Manag..

[18] Schultz G., Sibbald R.G., Falanga V., Ayello E.A., Dowsett C., Harding K., Romanelli M., Stacey M.C., Teot L., Vanscheidt W. (2003). Wound bed preparation: A systematic approach to wound management. Wound Repair Regen..

[19] Ayello E.A., Cuddigan J.E. (2004). Debridement: Controlling the necrotic/cellular burden. Adv. Skin Wound Care.

[20] Sen C.K., Roy S., Mathew-Steiner S., Gordillo G.M. (2021). Biofil management in wound care. Plast. Reconstr. Surg..

[21] De Francesco F., Marchesini A., Campodonico A., Neuendorf A.D., Pangrazi P.P., Riccio M. (2020). A multistep iter for functional reconstruction in mangled upper limb: A retrospective analysis of integrated surgical and medical approach. Medicina.

[22] Moog P., Jensch M., Betzl J., Bauer A.T., Cerny M.K., Schmauss D., Kukrek H., Erne H., Machens H.G., Megerle K. (2021). Bacterial bioburden of wounds: Influence of debridement and negative-pressure wound therapy (NPWT). J. Wound Care.

[23] Kravitz S.R., McGuire J., Zinszer K. (2008). Management of skin ulcers: Understanding the mechanism and selection of enzymatic debriding agents. Adv. Skin Wound Care.

[24] Falanga V. (2000). Classifications for wound bed preparation and stimulation of chronic wounds. Wound Repair Regen..

[25] Shah D., Mital K. (2018). The role of trypsin: Chymotrypsin in tissue repair. Adv. Ther..

[26] Forsling E. (1988). Comparison of saline and streptokinase-streptodornase in the treatment of leg ulcers. Eur. J. Clin. Pharm..

[27] Karlsson C., Andersson M.L., Collin M., Schmidtchen A., Bjorck L., Frick I.M. (2007). SulfA—A novel subtilisin-like serine proteinase of *Finegoldia magna*. Microbiology.

[28] Pavan R., Jain S., Shraddha, Kumar A. (2012). Properties and therapeutic application of bromelain: A review. Biotechnol. Res. Int..

[29] Ramundo J., Gray M. (2008). Enzymatic wound debridement. J. Wound Ostomy Cont. Nurs..

[30] Wright J.B., Shi L. (2003). Accuxyme (R) papain-urea debriding ointment: A historical review. Wounds.

[31] Marazzi M., Stefani A., Chiaratti A., Ordanini M.N., Falcone L., Rapisarda V. (2006). Effect of enzymatic debridement with collagenase on acute and chronic hard-to-heal wounds. J. Wound Care.

[32] Cortivo R., Abatangelo A. (2011). Bionect Start: The biological synergy for the evolution of enzymatic debridement. J. Wound Technol..

[33] Onesti M.G., Fioramonti P., Fino P., Sorvillo V., Carella S., Scuderi N. (2016). Effect of enzymatic debridement with two different collagenases versus mechanical debridement on chronic hard-to.-heal wounds. Int. Wound J..

[34] Mast B.A., Diegelmann R.F., Krummel T.M., Cohen I.K. (1993). Hyaluronic acid modulates proliferation, collagen and protein synthesis of cultured fetal fibroblasts. Matrix.

[35] Croce M.A., Dyne K., Boraldi F., Quaglino D., Cetta G., Tiozzo R., Pasquali Ronchetti I. (2001). Hyaluronan affects protein and collagen synthesis by in vitro human skin fibroblasts. Tissue Cell.

[36] Falanga V. (2008). Measurement in wound healing. Int. J. Low. Extrem. Wounds.

[37] Gravante G., Sorge R., Giordan N., Georgescu S.R., Morariu S.H., Stoicescu I., Clatici V. (2013). Multicenter clinical trial on the performance and tolerability of the Hyaluronic acid-collagenase ointment for the treatment of chronic venous ulcers: A preliminary pilot study. Eur. Rev. Med. Pharm. Sci..

[38] Gupta S., Sagar S., Maheshwari G., Kisaka T., Tripathi S. (2021). Chronic wounds: Magnitude, socioeconomic burden and consequences. Wounds Asia.

[39] Madhok B.M., Vowden K., Vowden P. (2013). New techniques for wound debridement. Int. Wound J..

[40] Sinclair R.D., Ryan T.J. (1994). Proteolytic enzymes in wound healing: The role of enzymatic debridement. Australas. J. Derm..

[41] McCallon S.K., Weir D., Lantis J.C. (2015). Optimizing wound bed preparation with collagenase enzymatic debridement. J. Am. Coll. Clin. Wound Spec..

[42] Di Pasquale R., Vaccaro S., Caputo M., Cuppari C., Caruso S., Catania A., Messina L. (2019). Collagenase-assisted wound bed preparation: An in vitro comparison between *Vibrio alginolyticus* and *Clostridium histolyticum* collagenases on substrate specifity. Int. Wound J..

[43] Ghosh K., Ren X.D., Shu X.Z., Prestwich G.D., Clark R.A.F. (2006). Fibronectin functional domains coupled to hyaluronan stimulate adult human dermal fibroblast responses critical for wound healing. Tissue Eng..

[44] Onesti M.G., Fioramonti P., Carella S., Fino P., Sorvillo V., Scuderi N. (2013). A new association between hyaluronic acid and collagenase in wound repair: An open study. Eur. Rev. Med. Pharm. Sci..

[45] Scalise A., Campitiello F., Della Corte A., Longobardi P., Di Salvo M., Tartaglione C., Santin C., Giordan N., Guarnera G. (2017). Enzymatic debridement: Is HA-collagenase the right synergy? Randomized double-blind controlled clinical trial in venous leg ulcers. Eur. Rev. Med. Pharm. Sci..

[46] Patry J., Blanchette V. (2017). Enzymatic debridement with collagenase in wounds and ulcers: A systematic review and meta-analysis. Int. Wound J..

[47] Foschi D., Castoldi L., Radaelli E., Abelli P., Calderini G., Rastrelli A., Mariscotti C., Marazzi M., Trabucchi E. (1990). Hyaluronic acid prevents oxygen free-radical damage to granulation tissue: A study in rats. Int. J. Tissue React..

[48] Riccio M., Marchesini A., Senesi L., Skrami E., Gesuita R., De Francesco F. (2019). Managing pathologic scars by injecting auto-cross-linked hyaluronic acid. A preliminary prospective clinical study. Aesthetic Plast. Surg..

